# Mapping the Functional Epitopes of Human Growth Hormone: Integrating Structural and Evolutionary Data with Clinical Variants

**DOI:** 10.3390/cimb47121012

**Published:** 2025-12-03

**Authors:** Sonia Verma, Amit V. Pandey

**Affiliations:** 1 Pediatric Endocrinology Unit, Department of Pediatrics, University Children’s Hospital Bern, 3010 Bern, Switzerland; soniaverma.jnu@gmail.com; 2 Translational Hormone Research Program, Department of Biomedical Research, University of Bern, 3010 Bern, Switzerland

**Keywords:** growth hormone, GH1, IGHD, growth disorders, human genome variation, population genetics, structural analysis

## Abstract

Human growth hormone (GH) exerts its pleiotropic effects by binding to its receptor (GHR), leading to receptor dimerization and activation. We combined structural, evolutionary, and genetic analyses to elucidate the critical determinants of GH-GHR interaction and the impact of disease-causing mutations. Protein contact analysis revealed the specific amino acid residues involved in two distinct binding interfaces between GH and two chains of GHR. ConSurf analysis demonstrated significant sequence conservation in the receptor-binding regions of GH across species, highlighting their functional importance. A comprehensive list of known disease-causing mutations in GH was compiled and mapped to these binding interfaces and conserved regions. Computational site-directed mutagenesis (SDM) analysis predicted the impact of several mutations on protein stability, revealing both stabilizing and destabilizing effects. Sequence comparisons with orthologs from various species further supported the evolutionary conservation of key functional residues. Integrated analysis of contact residues between GH and GHR showed a strong correlation between receptor-binding residues, evolutionary conservation, and the occurrence of disease-associated mutations. These findings underscore the critical role of specific GH residues in mediating high-affinity interactions with its receptor and how mutations in these conserved contact points can disrupt binding affinity and/or protein stability, ultimately leading to growth disorders. This multi-faceted approach provides valuable insights into the molecular mechanisms underlying growth hormone deficiency and related syndromes.

## 1. Introduction

Human growth and development is a highly regulated process governed by a complex network of steroid and peptide hormones. Steroid hormones, encompassing androgens (e.g., testosterone) and estrogens (e.g., estradiol), fulfill a critical role in growth, notably during puberty. These hormones contribute to the pubertal growth acceleration, facilitate bone maturation and epiphyseal closure (ultimately terminating linear growth), and are responsible for the development of secondary sexual characteristics and defect in genes encoding enzymes linked to steroid production lead to growth and developmental disorders [1,2,3,4,5]. Human growth hormone (GH), a peptide hormone, is critical in the complex regulation of growth, development, and metabolic processes throughout the human lifespan [6].

The gene encoding human growth hormone (GH1) is located on the long arm of chromosome 17, specifically at the 17q22-24 region [7]. This gene resides within a cluster of five closely related genes, including chorionic somatomammotropin hormone 1 (CSH1), CSH2, CSH-like 1 (CSHL1), and growth hormone variant 2 (GH2). This gene cluster spans approximately 65 kilobases [8]. The mature hGH protein, comprising 191 amino acids, is derived from a larger 217-amino acid precursor through the cleavage of a 26-amino acid signal peptide [9,10,11]. The three-dimensional structure of hGH is characterized by a distinctive four-helix bundle motif, a structural arrangement crucial for its interaction with the GHR, stabilized by two intramolecular disulfide bonds (Cys53–Cys165, Cys182–Cys189) [12]. Comparative analysis reveals significant conservation of the hGH amino acid sequence across species, particularly within regions involved in GHR binding.

Synthesis and secretion of GH are regulated by somatotrophic cells located in the anterior pituitary gland [8,9]. The pituitary gland is under the direct influence of the hypothalamus which plays a central role through GHRH, which stimulates GH release, and somatostatin, which inhibits it. Ghrelin, produced in the gastrointestinal tract, also promotes GH production and release [13,14]. The secretion of GH follows a pulsatile pattern, with the most significant release occurring during slow-wave sleep [15,16]. Various factors influence this release, including circadian rhythm, sleep–wake cycles, stress levels, physical exercise, and nutritional status [17,18,19,20,21]. Following secretion, GH circulates in the bloodstream primarily bound to a growth hormone-binding protein (GHBP), which is essentially the cleaved extracellular domain of the GHR [22,23]. GHBP acts as an GH reservoir and may modulate GH signaling. Its production is upregulated by GH, suggesting a negative feedback mechanism [24].

The secretion of GH is pulsatile, with a significant surge during deep sleep phases, particularly slow-wave sleep [25]. However, the efficacy of this hormone is entirely predicated on its structural fidelity. The biological activity of GH is mediated exclusively through its engagement with the transmembrane Growth Hormone Receptor (GHR), a member of the cytokine receptor superfamily. Unlike simple ligand-receptor pairs, the activation of the GHR signaling machinery requires a precise, sequential dimerization event. A single GH molecule functions as a bivalent ligand, utilizing two asymmetric binding surfaces, designated Site 1 and Site 2, to bridge two identical GHR subunits [26,27]. This dimerization is the molecular switch that brings the intracellular domains of the receptor into proximity, allowing the associated Janus Kinase 2 (JAK2) enzymes to trans-phosphorylate and initiate the Signal Transducer and Activator of Transcription 5 (STAT5) cascade. Consequently, the functional epitope of GH is not a single entity but a complex landscape of discontinuous amino acid residues distributed across its four-helix bundle architecture. The integrity of these interfaces is non-negotiable; structural perturbations can decouple binding from signaling [28]. Disruptions in the intricate GH signaling pathway, often due to mutations in the GH1 gene, can lead to various growth disorders [29,30,31]. Isolated growth hormone deficiency (IGHD), characterized by insufficient GH production, results in short stature and metabolic abnormalities [32,33,34,35,36]. IGHD can arise from mutations in either the GH1 gene or the growth hormone-releasing hormone and its receptor (GHRH and GHRHR) genes [37]. Several types of IGHD exist, each with distinct genetic underpinnings and varying severity [6,38]. Laron syndrome represents a primary form of GH insensitivity caused by GHR gene mutations. Mutations within the GH1 gene can lead to reduced hGH secretion or the production of variant GH proteins with impaired receptor binding or compromised signaling capabilities.

The clinical spectrum of *GH1* gene mutations reflects this structural complexity. While some mutations result in a complete absence of the hormone (Isolated Growth Hormone Deficiency, IGHD), others produce a ‘bioinactive’ molecule, a mutant hormone that circulates at normal concentrations and binds the receptor but fails to trigger the conformational changes necessary for signal transduction. Understanding the molecular pathology of these variants requires moving beyond simple genetic identification to a multi-dimensional analysis of the protein’s biophysics. By mapping clinical variants onto the evolutionary landscape of the hormone and modeling their thermodynamic impact on the GH-GHR interface, we can elucidate the distinct mechanisms, whether protein instability, Site 1 disruption, or Site 2 blockade, that underlie phenotypic diversity in growth disorders.

## 2. Materials and Methods

### 2.1. Sequence Alignments

The CLC Protein Workbench (available at: https://digitalinsights.qiagen.com/products-overview/discovery-insights-portfolio/analysis-and-visualization/qiagen-clc-main-workbench/, accessed on 20 November 2025) was used for multiple sequence alignments and pairwise comparisons to calculate sequence identity and similarity percentages reported in Section 3.1, employing standard settings. The Multiple Sequence alignment included sequences of growth hormones from *Homo sapiens* (P01241), *Macaca mulatta* (P33093), *Rattus norvegicus* (P01244), *Mus musculus* (P06880), *Equus caballus* (P01245), *Sus scrofa* (P01248), *Bos Taurus* (P01246), *Ovis aries* (P67930), *Cavia porcellus* (Q9JKM4), *Meleagris gallopavo* (P22077), *Gallus gallus* (P08998), *Struthio camelus* (Q9PWG3), *Anguilla japonica* (P08899), *Carassius auratus* (O93359), and *Salmo salar* (Q5SDS1).

### 2.2. Evolutionary Conservation of Amino Acids

The HGH protein sequence (NP_000506.2) was used as a query sequence to search the homologous sequences in the Uniref90 database by performing a PSI-Blast search using default settings [39]. To rigorously assess the evolutionary constraints acting on the mature GH molecule, the ConSurf server (https://consurf.tau.ac.il/, accessed on 20 November 2025) was employed using the Bayesian inference method. This probabilistic approach is superior for smaller datasets as it accounts for the stochastic nature of the evolutionary process and the phylogenetic relationships between sequences. A multiple sequence alignment (MSA) of orthologous vertebrate GH sequences was constructed using MAFFT, and the evolutionary rates were calculated based on the JTT amino acid substitution matrix. Conservation scores were normalized to a 9-point scale, where 1 denotes maximal variability and 9 denotes maximal conservation, with confidence intervals calculated for each position to ensure statistical reliability [40].

### 2.3. Protein Structure Loop Modeling

HGH crystal structure solved with its receptor (PDB Id: 3HHR, dimeric receptor) [12] has few missing residues in the loop regions (Chain A: 148–154; Chain B & C: 57–62, 73–78), those were modeled by using loop modeling module of YASARA (version 25.9.17, www.yasara.org) [41]. For each loop region, ten loop conformations were generated and out of those, an energy minimized conformation was selected for the loop replacement. The structure models were visualized by Pymol (version 3.1.6.1, www.pymol.org) and rendered as ray-traced images with POVRAY (version 3.7.0.10, www.povray.org).

### 2.4. HGH-Receptor Interaction Analysis

HGH structure (PDB Id: 3HHR, Chain A: HGH, Chains B & C: Receptor) was analyzed to find the residues, in contact with its receptor via covalent or non-covalent bonding. The PDBsum Generate service (available at: https://www.ebi.ac.uk/thornton-srv/databases/pdbsum/Generate.html, accessed on 20 November 2025) was used for creating the contact map, with default parameters for identifying hydrogen bonds and non-bonded contacts [12].

### 2.5. Disease-Causing Mutations

The data related to disease-causing mutations were obtained from UniProtKB [42]. UniProtKB compiles disease-causing mutation data related to a protein under-involvement in the disease section.

### 2.6. Prediction of Protein Stability upon Mutation Using Site-Directed Mutagenesis Tool (SDM)

The thermodynamic impact of *GH1* missense mutations was quantified using the Site Directed Mutator (SDM) server. This algorithm predicts the change in free energy of unfolding (ΔΔG) between the wild-type and mutant structures [43,44]. The crystal structure of the GH-GHR complex (PDB ID: 3HHR) served as the structural template. To isolate the intrinsic stability of the hormone, Chain A (GH) was analyzed independently of the receptor chains. The algorithm utilizes environment-specific substitution tables—calculated from a database of homologous protein families—to estimate the energetic penalty of introducing a specific amino acid side chain into a defined local environment (secondary structure, solvent accessibility, and hydrogen bonding network). A negative ΔΔG value indicates a destabilizing mutation (increased free energy of the folded state), while a positive value suggests a stabilizing effect. Mutations with ΔΔG < −1.0 kcal/mol were classified as highly destabilizing, correlating with likely defects in protein folding or secretion [45,46].

### 2.7. Analysis of HGH Allele Frequencies from Genome Sequencing

Frequencies from Exome Aggregation Consortium [47], NHLBI Exome Sequencing Project (NHLBI Exome Sequencing Project, 2016), and 1000 Genomes [48] were collected and assigned to the main categories of the 1000 Genomes project.

## 3. Results

### 3.1. Analysis of GH Sequence Across Species

The sequence comparison reveals varying degrees of identity and similarity between human growth hormone and its orthologs in different species (Table 1). As expected, the highest sequence identity (96%) and similarity (97%) are observed with Rhesus macaque, a closely related primate. Among the other mammalian species examined, sequence identity to human hGH ranges from 65% to 68%, with sequence similarity ranging from 76% to 79% in rat, mouse, horse, pig, bovine, sheep, and guinea pig. The avian species, common turkey and chicken, show lower sequence identity (55% and 57%, respectively) and similarity (73% and 74%, respectively) to human hGH. The common ostrich exhibits similar values (54% identity and 72% similarity). The lowest sequence identity and similarity are observed in the fish species. Japanese eel shows 44% identity and 61% similarity, goldfish has 38% identity and 58% similarity, and Atlantic salmon displays the lowest values with 36% identity and 52% similarity to human GH.

The length of the mature growth hormone protein is highly conserved across the mammalian species, ranging from 216 to 217 amino acids, similar to the 217 amino acids in human hGH. The signal peptide length is also conserved at 26 amino acids in most mammals, with a slightly shorter signal peptide of 25 amino acids in the avian species and 19–22 amino acids in the fish species.

#### 3.1.1. Analysis of Human GH Mutations and Their Locations in GH Protein

**Figure 1:**
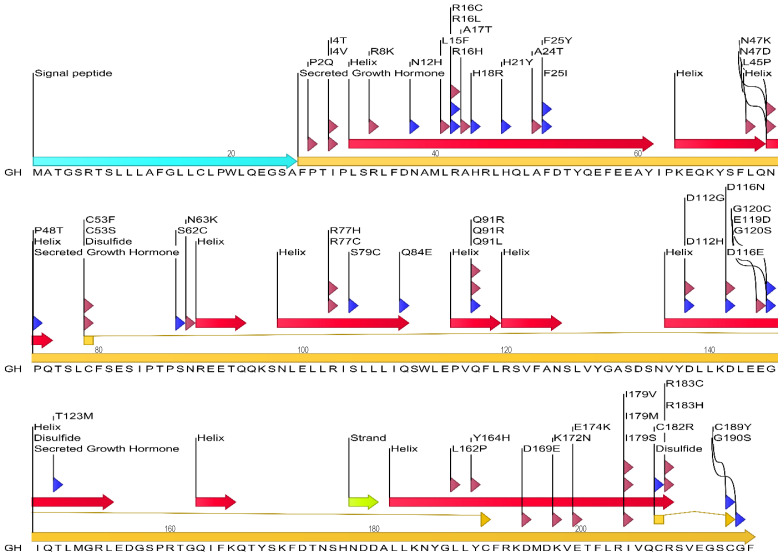
Schematic representation of the human growth hormone protein sequence and location of common mutations. The linear sequence of the secreted 217 amino acid GH protein is shown, indicating key secondary structure elements (α-helices represented by red arrows, β-strand by a yellow arrow, and disulfide bonds by connecting lines). The signal peptide (26 amino acids) is shown at the N-terminus, followed by the mature secreted GH. Various reported mutations are indicated above and below the sequence, with arrows pointing to the affected amino acid residue. Mutations associated with growth hormone deficiency (GHD) or Laron syndrome (LS) are highlighted. The figure illustrates the distribution of these mutations across the GH protein, including within and outside the α-helical regions known to be crucial for interaction with the growth hormone receptor.

We first performed an analysis of the human GH protein sequence and the location of numerous reported mutations (Figure 1). Several key structural features of GH are marked in Figure 1, including four major α-helices (A, B, C, and D) and two disulfide bonds connecting cysteine residues at positions 53–165 and 182–189. Several point mutations are depicted along the linear sequence, distributed throughout the protein. A significant number of mutations are observed within the α-helical regions, particularly in helix A (residues 9–34) and helix D (residues 158–190), which are known to be critical for binding to the growth hormone receptor (GHR). For instance, mutations like R16C, R16L, R16H, and A17T are located in helix A. Similarly, mutations such as F25Y and F25I are also found in this region. In helix D, mutations like Y164H, K172N, E174K, I179V, I179M, I179S, C182R, R183C, R183H, C189Y, and G190S are present. Mutations are also observed in the loop regions connecting the helices. For example, P2Q, I4T, I4V, R8K, N12H, L15F, H18R, H21Y, A24T, N47K, N47D, and L45P are located in the N-terminal region and the loop between helix A and B. Mutations like C53F, C53S, N63K, S62C, R77H, R77C, S79C, Q84E, Q91R, and Q91L are found in the loop connecting helix B and C, and within helix C (residues 97–107). In the C-terminal region and the loop between helix C and D, mutations such as T123M, L162P, D169E, D112G, D112H, D116N, D116E, G120C, G120S, and E119D are indicated.

Known GH disease-causing mutation data were retrieved from the UniProtKB database (Table 2). GH mutations are found to be responsible for two types of diseases: Isolated growth hormone deficiency (GHD) and Kowarski syndrome (KWKS). The growth hormone deficiency is an autosomal recessive deficiency of growth hormone leading to short stature. Patients have low but detectable levels of growth hormone, significantly retarded bone age, and a positive response and immunologic tolerance to growth hormone therapy. The Kowarski syndrome is clinically characterized by short stature associated with bio-inactive growth hormone, normal or slightly increased growth hormone secretion, pathologically low insulin-like growth factor 1 levels, and normal catch-up growth on growth hormone replacement therapy. These mutations are distributed throughout the GH protein and exhibit diverse effects on its functional activity. Certain mutations, including L16P and Q117L, predominantly compromise GH secretion. Conversely, mutations such as T53I, K67R, N73D, S97F, and T201A hinder the capacity of GH to activate the JAK/STAT signaling pathway. Finally, the R209H mutation is associated with autosomal dominant growth hormone deficiency (IGHD2). The HGH variants Thr3, Arg16, Asn47, Gln91, Arg183 and Arg77, Asp112 are associated with growth hormone deficiency [49,50,51] and Kowarski syndrome [50,52,53,54], respectively. It is noteworthy that the R103C mutation, implicated in Kowarski syndrome, does not impede GHR signaling or interaction, but rather demonstrates an augmented interaction with GHBP. The D138G mutation, also associated with KWKS, results in an abrogation of biological activity.

#### 3.1.2. Comparison of Human GH Protein Sequence with Diverse GH Homologues Across Species

**Figure 2:**
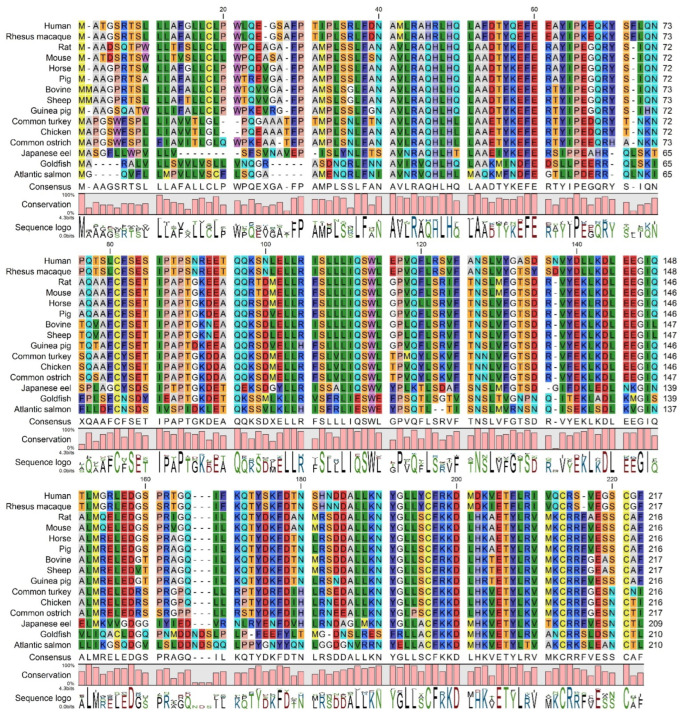
A multiple sequence alignment of human growth hormone alongside growth hormone sequences from various vertebrate species. The alignment encompasses the N-terminal signal peptide, where applicable, and the mature growth hormone sequence. The represented species, arranged from top to bottom, are as follows: Human, Rhesus macaque, Rat, Mouse, Horse, Pig, Bovine, Sheep, Guinea pig, Common turkey, Chicken, Common ostrich, Japanese eel, Goldfish, and Atlantic salmon. Residues are color-coded based on their chemical characteristics to accentuate patterns of conservation. The consensus sequence and conservation levels are presented beneath the alignment. Notable highly conserved residues and regions are observed throughout the mature growth hormone sequence, indicating their potential functional significance.

**Figure 3:**
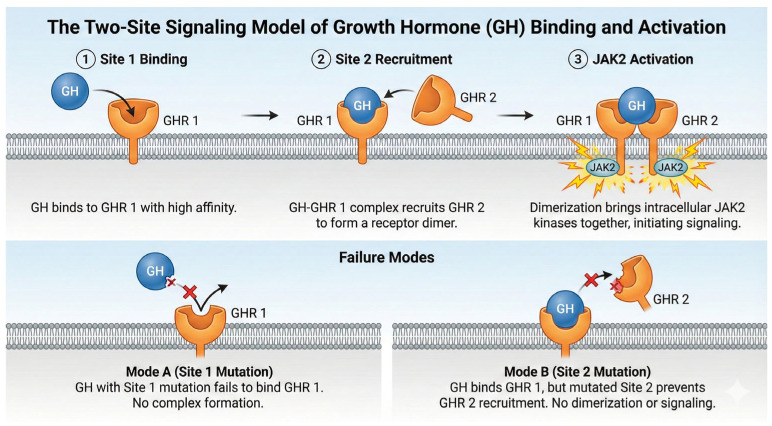
Mechanistic Divergence of Signaling Failure. A schematic illustration of the sequential dimerization model. Wild-type signaling (**top**) requires successful engagement of both Site 1 and Site 2 to dimerize the receptor and activate JAK2. Site 1 mutations (**bottom left**) prevent initial capture. Site 2 mutations (**bottom right**), typical of bioinactive GH, allow initial binding but block dimerization, creating an inert 1:1 complex that acts as a competitive inhibitor of signaling.

A multiple sequence alignment (Figure 2) analysis demonstrated a high degree of conservation within the full as well as mature growth hormone sequence across a diverse array of vertebrate species, while the N-terminal signal peptide exhibits increased variability. This pattern of conservation and variability is characterized by three principal observations. **1. Extensively Conserved Domains:** Discrete segments of amino acids within the mature growth hormone sequence are nearly invariant across mammalian species and demonstrate considerable conservation in more phylogenetically distant species, such as avian and piscine taxa. These extensively conserved domains likely represent critical structural components or functional regions of the growth hormone protein essential for its biological activity. Any alterations within these domains may potentially disrupt the protein’s structural integrity or functional capacity, resulting in deleterious consequences. **2. Invariant Residues:** The alignment also reveals numerous individual amino acid residues that are completely conserved across the majority, if not all, of the aligned sequences. These conserved residues may encompass cysteine residues implicated in disulfide bridge formation, proline residues critical for secondary structure stabilization, or other residues involved in receptor binding or protein–protein interactions (Figure 3). The absolute conservation of these specific residues throughout evolutionary time strongly suggests their indispensable roles in maintaining the protein’s structural framework, stability, receptor affinity, or other essential functions. **3. Variable Segments:** Compared to the highly conserved domains, specific segments within the mature growth hormone sequence exhibit greater variability, particularly when comparing sequences from distantly related species. These variable segments may be involved in species-specific adaptations, facilitating interactions with disparate receptors or responses to divergent physiological conditions. Additionally, these segments may possess greater tolerance to amino acid substitutions without inducing substantial functional impairment.

### 3.2. Analysis of GH-GHR Contacts

#### 3.2.1. Structural Architecture of the High-Affinity Interface (Site 1)

**Figure 4:**
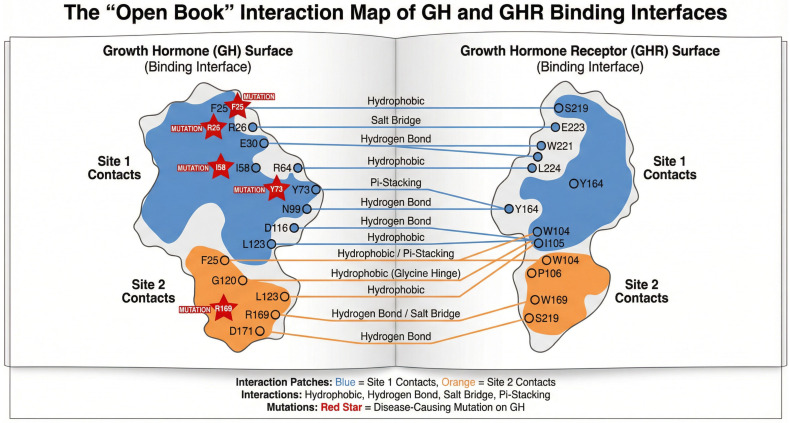
Schematic Interaction Map of the GH-GHR Interface. An ‘open-book’ view of the complementary surfaces involved in signaling. The High-Affinity Site 1 (blue) relies on a hydrophobic core centered on Phe25, while the Low-Affinity Site 2 (orange) engages the N-terminal and Helix C regions. Disease-associated residues (marked with red stars) predominantly cluster within these contact zones, illustrating that pathogenicity is frequently driven by the disruption of specific interface ‘hotspots’ rather than global structural failure.

The primary recruitment of the receptor is mediated by a large, concave surface on GH (Site 1) comprising residues from Helix A and Helix D. Our contact analysis (Figure 4) identifies this interface as an “affinity trap,” stabilized by a hydrophobic core surrounded by a ring of electrostatic interactions. The central hydrophobic patch is anchored by Phe25, Leu45, Pro48, and Ile179, which bury their side chains into the receptor’s binding pocket. This hydrophobic collapse is complemented by a specific network of hydrogen bonds that are likely to provide specificity, including His18-Arg217, Phe25-Ser219, and Glu174-Lys167. Notably, the interaction between Arg167 on GH and Glu127 on the receptor suggests an electrostatic steering mechanism that guides the initial docking trajectory.

Mutations mapping to this surface, such as K172N and E174K, directly perturb this binding network. While these variants may allow the protein to fold correctly, they compromise the obligate first step of receptor capture, resulting in a circulating hormone that is immunologically detectable but biologically impotent.

#### 3.2.2. The Dimerization Interface (Site 2) and Signaling Activation

The formation of the active signaling complex requires the recruitment of a second GHR molecule to Site 2, a topologically distinct surface formed by the N-terminus and Helix C (Figure 4). In contrast to Site 1, this interface is smaller and flatter, relying on critical contacts involving Phe1, Ile4, and Arg16 at the N-terminus, and Asp116, Glu119, and Gly120 on Helix C.

The biological necessity of this interface is highlighted by the “bioinactive” phenotype observed in Kowarski syndrome. Variants such as D116E and G120C map precisely to this surface. Our analysis shows that Gly120 sits in a sterically restricted region of the helix; substitution with a bulkier cysteine or arginine side chain creates steric clashes that physically block the approach of the second receptor. Because Site 1 remains intact, these mutants act as competitive antagonists: they occupy the receptor but fail to trigger the dimerization-induced conformational change required for JAK2 phosphorylation.

#### 3.2.3. Cross-Talk and Overlapping Residues

Interestingly, specific residues such as Arg16 and Thr123 appear to participate in the structural organization of both interfaces or lie at the pivot point between them. Arg16, located on the loop connecting Helix A, forms a hydrogen bond with Glu44 of the second receptor chain while maintaining proximity to the Site 1 interface. This dual involvement suggests that the N-terminal region may act as a structural hinge, coupling the binding events at Site 1 with the conformational adjustments required for Site 2 engagement.

The mapping of known disease mutations to these interfaces provides direct structural explanations for their functional consequences. For instance, mutations at residues like R16 (R16C/L/H), H18 (H18R), and H21 (H21Y), which are involved in GHR contacts (Figure 4), are associated with GHD. The R16C mutation, specifically, is linked to reduced secretion and IGHD1B, consistent with its location at a key interface residue interacting with Glu44 on GHR chain C and proximity to the chain B interface. Similarly, mutations like D116N/E and G120C/S, impacting the second GHR binding site (Chain C) and associated with IGHD1B or Kowarski Syndrome (D112G/H near this site), likely disrupt receptor dimerization essential for signaling.

Comparison with our contact analysis (Table 3 and Figure 4) reveals a significant overlap in the identified contact residues for both GHR chains. Most of the GH residues listed as contacting either chain B or chain C in this table were also highlighted in the respective contact figures, validating the consistency of the contact mapping.

### 3.3. Analysis of Sequence Conservation Pattern in Growth Hormone at GH-GHR Contact Points

**Figure 5:**
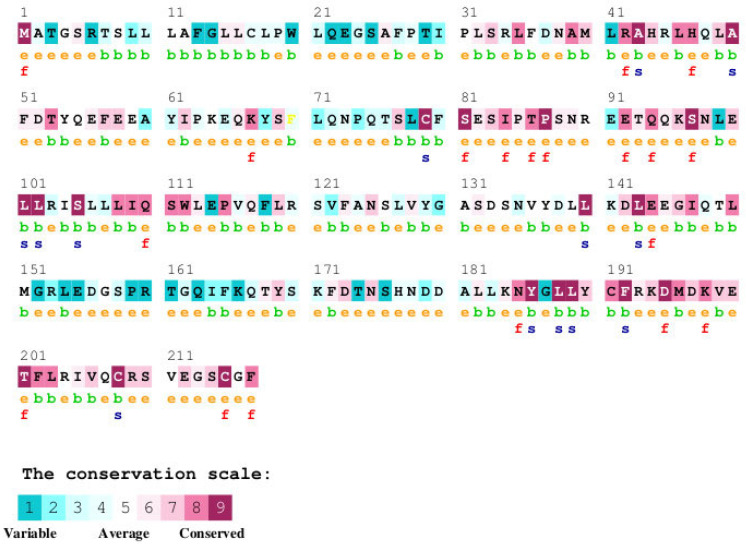
ConSurf analysis of the human growth hormone (hGH) protein showing sequence conservation. The amino acid sequence of hGH is displayed, with each residue colored according to its conservation level, ranging from variable (cyan to blue) through average (green) to conserved (yellow to maroon). The conservation scores are based on a multiple sequence alignment of hGH orthologs from various species. Predicted functional residues (highly conserved and exposed) are marked with an ‘f’, and predicted structural residues (highly conserved and buried) are marked with an ‘s’. Exposed residues are also indicated with an ‘e’, and buried residues with a ‘b’.

Analysis of the ConSurf conservation scores for these contact residues shows a general trend towards higher conservation. Many of the GH residues involved in receptor binding exhibit scores of 6 or higher, indicating their evolutionary importance (Figure 5). For instance, residues such as R16, H18, H21, F25, Y42, Y47, F51, L45, P48, S62, N63, Q91, Y103, D116, E119, T123, Y164, R167, K172, E174, R178, I179, C182, C189, G190, and F1, P2, I4, R8, N12, L15, L9, G120, and T123 (contacting chain C) display notable conservation. Furthermore, several GH residues that are known to be mutated in human diseases (UniProt Disease Variant column) are also present in Table 2. Many of these clinically significant residues, such as P2, I4, R8, N12, L15, R16 (R>C), A17, H18, H21, A24, F25, L45, N47 (N>D), P48, S62, N63, Q91, D112, D116, E119, G120, T123, L162, Y164, D169, K172, E174, I179, C182, R183, C189, and G190, are also identified as contact residues for one or both chains of the GHR.

The ConSurf analysis (Figure 5, Table 4) reveals a significant degree of sequence conservation across the human growth hormone protein, with several regions exhibiting high conservation scores. The N-terminal region of the mature hGH (residues 1~40) shows a mix of conservation levels, with some highly conserved patches interspersed with more variable residues. Notably, residues R16, H18, H21, and F25 within this region, which are known to be involved in receptor binding (as shown in Figure 3 and Figure 4), display high conservation. The region encompassing helix C and the loop connecting it to helix D (approximately residues 95–145) also shows considerable conservation, with several residues predicted to be functional (‘f’) or structural (‘s’). Specifically, residues like Q91, Y103, and D116, which participate in receptor binding, are located within this conserved segment (Figure 2). The C-terminal helix D (residues ~158–190), another crucial region for receptor interaction, exhibits a high degree of conservation. Residues Y164, R167, K172, E174, R178, I179, C182, C189, and G190, all implicated in binding to the growth hormone receptor (Figure 2), are located within this highly conserved helix. Several of these are also predicted to be functional (‘f’) or structural (‘s’). In contrast, some loop regions and the very C-terminus (residues 191–217) appear to be more variable, suggesting less stringent evolutionary constraints on these parts of the protein.

The ConSurf analysis highlights that many residues critical for GHR interaction are highly conserved across species (Figure 5), underscoring their functional importance. Residues like Cys53 and Cys182, involved in disulfide bonds and showing the highest conservation score (9), are crucial for structural integrity. Mutations like C53S/F are predicted to be destabilizing (Table 5) and are linked to GHD, emphasizing the importance of structural stability. Furthermore, many disease-associated mutations occur at highly conserved contact residues, such as R16 (score 7), H18 (score 7), H21 (score 7), K172 (score 9), E174 (score 9), and R183 (score 9), linking evolutionary pressure, structural role, and disease pathology. However, mutations in less conserved residues like D112 (score 1), which causes Kowarski Syndrome (D112G), indicate that variability does not preclude functional importance, possibly in subtle conformational dynamics or specific interactions.

### 3.4. Stability Analysis of GH Mutations

All selected HGH variants from previous analyses were subjected to site-directed mutagenesis via the SDM tool (Table 5). SDM software (version 6.23) predicts the effect of the mutation on protein stability and potentiality as a disease-causing mutant. SDM uses a set of conformationally constrained environment-specific scoring matrices to calculate the difference in stability (pseudo ∆∆G score) between the wild-type and mutant protein upon providing a structure of wild-type and a list of mutations [43,44]. The negative and positive values of pseudo ∆∆G correspond to mutations predicted to be destabilizing and stabilizing, respectively. Besides the pseudo ∆∆G score SDM output also provide information about various structural features, including class of secondary structure (SSE), solvent accessibility (RSA), residue depth (DEPT), occluded surface packing (OSP) sidechain-sidechain hydrogen bonding (SS), sidechain-mainchain amide hydrogen bonding (SN) and sidechain-mainchain carbonyl hydrogen bonding (SO) for the wild-type and mutant residues [44]. Previously, it has been predicted that the highly destabilizing mutations are mostly found at high residue packing density regions (OSP > 0.56) and occur at two distinct depth levels (4 Å and 8 Å) and highly stabilizing mutations were observed to occur mostly at high packing density regions and residue depth ∼4 Å [44].

**Figure 6:**
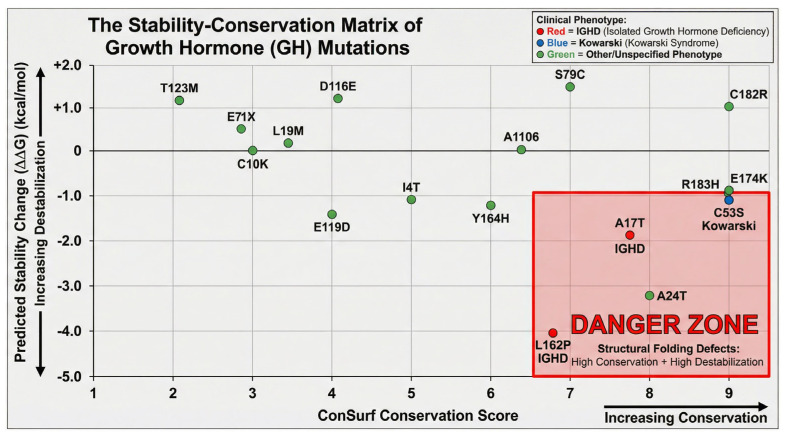
The Evolutionary Thermodynamic Landscape of *GH1* Variants. This matrix correlates the evolutionary importance of a residue (*X*-axis) with the thermodynamic consequence of its mutation (*Y*-axis). Variants falling into the ‘Structural Folding Defect’ zone (**bottom right**) are highly conserved residues that suffer massive destabilization upon mutation (e.g., L162P), leading to secretory failure. Conversely, variants in the ‘Functional Defect’ zone (**top right**) are highly conserved but stable, indicating a direct loss of receptor binding function without protein unfolding.

In our analysis, we have found that out of 51 HGH variants 31 are responsible for the reduced stability of the protein. The Site-Directed Mutagenesis (SDM) analysis predicted a range of stability changes (ΔΔG) for the selected mutations in the human growth hormone protein (Table 5, Figure 6). The predicted ΔΔG values ranged from −4.31 kcal/mol to 1.52 kcal/mol, indicating that some mutations are predicted to increase protein stability while others are predicted to decrease it.

Several mutations were predicted to significantly destabilize the protein (negative ΔΔG values). These include L162P (−4.31 kcal/mol), A24T (−3.21 kcal/mol), L45P (−2.23 kcal/mol), A17T (−1.88 kcal/mol), E119D (−1.48 kcal/mol), Y164H (−1.27 kcal/mol), I4T (−1.09 kcal/mol), C53S (−1.11 kcal/mol), R183H (−1.1 kcal/mol), and E174K (−1.01 kcal/mol). Conversely, some mutations were predicted to increase protein stability (positive ΔΔG values), such as S79C (1.52 kcal/mol), D116E (1.25 kcal/mol), T123M (1.19 kcal/mol), C182R (1.04 kcal/mol), D112H (0.88 kcal/mol), C189Y (0.78 kcal/mol), G120C (0.7 kcal/mol), N12H (0.68 kcal/mol), H21Y (0.65 kcal/mol), S62C (0.62 kcal/mol), F25Y (0.47 kcal/mol), Q84E (0.4 kcal/mol), R16L (0.39 kcal/mol), P48T (0.34 kcal/mol), F25I (0.31 kcal/mol), Q91L (0.29 kcal/mol), R16H (0.19 kcal/mol), G120S (0.18 kcal/mol), and H18R (0.06 kcal/mol).

Other mutations showed minimal predicted changes in stability, with ΔΔG values close to zero, such as I4V (−0.07 kcal/mol), R77H (−0.07 kcal/mol), D169E (−0.01 kcal/mol), and I179M (−0.02 kcal/mol). The HGH variants L162P and A17T scored higher pseudo ∆∆G values (−4.31 and −3.21, respectively) and are linked with growth hormone deficiency. Similarly, variant C53S (∆∆G −1.11) is linked with Kowarski syndrome.

## 4. Discussion

The sequence comparison of human growth hormone with its orthologs across a diverse range of species highlights the evolutionary conservation of this important hormone. The high degree of sequence identity and similarity observed in primates underscores the close evolutionary relationship and likely conservation of function. The moderate sequence identity and similarity in other mammalian species suggest a conserved core function of growth hormone, although some species-specific variations might exist, potentially reflecting adaptations to different physiological needs or receptor interactions. The lower sequence identity and similarity in avian and fish species indicate a greater evolutionary distance, which is expected given the phylogenetic relationships. However, the fact that significant sequence similarity is still present suggests that the fundamental roles of growth hormone have been maintained throughout vertebrate evolution.

Mutations within the highly conserved regions of the human growth hormone gene are more probable to impair the normal protein function. Such mutations may precipitate various growth disorders, including growth hormone deficiency or Laron syndrome. The specific conserved residues identified warrant further investigation, as mutations at these sites in human GH may have severe functional consequences, potentially affecting protein folding, secretion, receptor binding affinity, or downstream signaling. These findings align with the ConSurf analysis, where highly conserved regions in human growth hormone are likely to correspond to residues that are identical or highly similar in these other species. These conserved residues are likely crucial for maintaining the protein’s structure, stability, and interactions with the growth hormone receptor. The variations in sequence identity and similarity across species can also provide insights into which regions of the protein might be more amenable to change and potentially responsible for species-specific effects of growth hormone.

The spectrum of GH1 mutations leading to growth disorders underscores the critical importance of various structural and functional aspects of the growth hormone protein. Mutations affecting secretion likely disrupt the processes involved in GH synthesis, post-translational modification, or release from somatotroph cells in the anterior pituitary. Impairment of JAK/STAT pathway activation indicates that these mutations might affect the interaction of GH with its receptor (GHR) or the subsequent conformational changes required for downstream signaling. The unique case of the R103C mutation in Kowarski syndrome, which enhances GHBP interaction without affecting GHR binding or signaling, suggests a more complex regulatory role for GHBP in GH action or availability. The D138G mutation leading to a complete loss of activity likely results in a severely misfolded or non-functional GH protein. The identification of these and other mutations provides valuable insights into the genotype–phenotype correlations in growth hormone deficiency and related syndromes.

The clustering of mutations within the α-helical regions, particularly helix A and D, which are known to form the primary binding interface with the GHR, suggests that these mutations are highly likely to disrupt the crucial contacts required for receptor activation. Amino acid substitutions in these regions can alter the shape, charge, or hydrophobicity of the binding surface, potentially leading to reduced binding affinity or complete loss of interaction, as observed in some forms of growth hormone deficiency (GHD) and Laron syndrome (LS). Mutations in residues such as Asp116 or Glu119 on hGH, which contact GHR Chain C, can disrupt the receptor dimerization process. Even if the initial binding to the first receptor occurs, the failure to recruit and bind the second receptor molecule will prevent full receptor activation and downstream signaling. This can also lead to growth hormone resistance.

Mutations located in the loop regions or outside the main helical structures might affect protein folding, stability, or the subtle conformational changes necessary for optimal receptor binding. For instance, mutations near or within the disulfide bonds (C53-C165 and C182-C189) could disrupt the proper tertiary structure of GH, indirectly affecting its interaction with the GHR.

The diversity and widespread distribution of the identified mutations underscore the complexity of genetic factors influencing growth. While some mutations may lead to a complete absence of functional GH, others might result in the secretion of a structurally altered hormone with impaired receptor binding or signaling capabilities.

The interaction of GH with its receptor involves a two-site binding mechanism, where one molecule of GH sequentially binds to two receptor monomers, leading to receptor dimerization and activation. Our contact analysis reveals the distinct sets of amino acid residues on GH that mediate these two binding events. The first interface (GH-GHR chain B) involves a broad range of interactions across different helical regions of hGH. The second interface (GH-GHR chain C) involves a separate set of residues, particularly in the N-terminal region and helix C of GH. This kinetic disparity is functionally critical; the high affinity of Site 1 ensures rapid capture of circulating GH by surface receptors, while the lower affinity of Site 2 prevents premature dimerization or receptor sequestration at sub-physiological hormone concentrations.

The presence of several predicted functional (‘f’) and structural (‘s’) residues within these conserved regions further emphasizes their importance. Functional residues, being highly conserved and exposed, are likely involved in direct interactions, such as receptor binding. Structural residues, being highly conserved and buried, are crucial for maintaining the protein’s three-dimensional fold and stability, which indirectly supports proper receptor interaction.

The variability observed in some loop regions and the C-terminus might indicate that these regions are less critical for the core function of receptor binding or may be involved in more species-specific roles or interactions that do not necessitate high conservation across all orthologs. The presence of common mutations at the residues involved in both interfaces highlights the critical role of these regions in receptor binding and function. For example, mutations like R16C/L/H are located at a key residue that interacts with both receptor chains (Glu44 on chain C and is in proximity to the interface with chain B as seen in Figure 4). This suggests that mutations at this position are highly likely to disrupt the formation of the functional receptor dimer, further highlighting evolutionary pressure to maintain the integrity of these functional regions.

Similarly, mutations in the N-terminal region (e.g., P2Q, I4T/V, R8K, N12H, and L15F) affect the second binding site, potentially impairing the initial engagement or the subsequent dimerization step. Mutations in helix C (e.g., G120C/S, D116N/E, E119D, and T123M) also disrupt the second interface. The fact that different sets of mutations affect the two binding sites could have implications for the specificity and affinity of hGH for its receptor. Mutations affecting the first, higher-affinity binding site might have a more profound effect on overall receptor activation. However, disruptions in the second binding site, crucial for receptor dimerization, can also lead to significant impairments in growth hormone signaling. The strong overlap between the contact residues identified in this table and our contact analysis reinforces the accuracy of the interaction mapping.

The observation that many of the GH residues involved in contacting the receptor are highly conserved (as per ConSurf analysis) underscores the evolutionary pressure to maintain these crucial binding interfaces. These conserved residues likely play critical roles in the affinity and specificity of the GH-GHR interaction, which is essential for proper growth hormone signaling. The significant number of disease-associated mutations occurring at these contact residues highlights the direct link between disruptions in receptor binding and the development of growth disorders. Mutations in these key residues can potentially alter the conformation of GH at the binding interface, reduce its affinity for the receptor, or impair the formation of the functional receptor dimer.

The SDM stability predictions (Table 5) provide further mechanistic insights. Highly destabilizing mutations like L162P (ΔΔG −4.31), A17T (ΔΔG −1.88), and L45P (ΔΔG −2.23) occur in helical regions (Table 5) known to be important for structure and/or receptor binding (Site 1 for L45P, proximity for A17T). This predicted instability likely contributes to their association with GHD by affecting folding, secretion, or binding competence. For instance, the L162P mutation introduces a proline into helix D, likely disrupting the helical structure critical for receptor interaction. Proline residues act as ‘helix breakers’ because they lack the amide hydrogen required for the canonical alpha-helical hydrogen bonding network. The introduction of Proline at position 162 likely unwinds the C-terminal region of Helix D, destabilizing the four-helix bundle core and triggering quality control mechanisms that retain the misfolded protein in the endoplasmic reticulum.

Conversely, mutations predicted to increase stability, such as S79C (ΔΔG +1.52) or D116E (ΔΔG +1.25), might impair function by hindering necessary conformational flexibility for receptor activation or release. The D116E mutation, affecting the second GHR binding site, is linked to IGHD1B, suggesting stability changes can disrupt function. Integrating stability predictions with contact analysis and conservation data strengthens the genotype-phenotype correlation; for example, the R183H mutation (IGHD2) is highly conserved (score 9), located near the Cys182-Cys189 disulfide bond, involved in GHR contact (Table 3), and predicted to be significantly destabilizing (ΔΔG −1.1).

The analysis of the ConSurf conservation scores in relation to the selected GH mutations reveals a trend where many disease-associated mutations occur at residues that are highly conserved across species. This observation supports the idea that these conserved residues play crucial roles in the structure, function, or interactions of the growth hormone protein. Mutations at such positions are more likely to disrupt these critical aspects, leading to phenotypic consequences such as growth hormone deficiency. The evolutionary data unambiguously identifies the disulfide bridges as non-negotiable structural elements (ConSurf Score 9). The SDM analysis corroborates this, with mutations such as C53S and C182R consistently predicted to destabilize the protein fold (ΔΔG < −1.0 kcal/mol). Beyond simple stability, the disruption of these cysteines has profound implications for the mode of inheritance. In IGHD Type II, mutations often affect the C-terminal region or splicing, but missense mutations involving cysteine residues can induce a dominant-negative effect. If a mutation leaves a cysteine residue unpaired (e.g., C182R), the remaining free thiol at position 189 becomes a reactive nucleophile. This can lead to the formation of aberrant intermolecular disulfide bonds, cross-linking mutant GH with wild-type GH within the high-density environment of the secretory granule forming insoluble aggregates that deplete the pool of functional hormone, a mechanism characteristic of Autosomal Dominant IGHD Type II.

However, the presence of disease-associated mutations at less conserved positions, such as L15F and D112G/H, indicates that even variable residues can play a role in normal protein function, and changes at these sites can sometimes lead to disease. It is possible that these residues contribute to more subtle aspects of protein function or might be involved in interactions that are not universally conserved across all orthologs examined in the ConSurf analysis. The residue variety information provides additional context to the conservation scores. Positions with high conservation and low residue variety, like Cys53, likely have very specific structural or functional roles that cannot be easily substituted by other amino acids. Conversely, positions with low conservation and high residue variety, like Leu15, might be more tolerant to amino acid changes.

The results of the SDM analysis provide insights into the potential impact of genetic variations on the stability of the human growth hormone protein. Protein stability is crucial for proper folding, function, and interactions, and alterations in stability can lead to impaired biological activity and disease. The prediction that several disease-associated mutations destabilize the GH protein (e.g., L162P, A24T, L45P, A17T, E119D, Y164H) suggests that these mutations might disrupt the native conformation of the hormone, potentially affecting its binding affinity to the growth hormone receptor or its downstream signaling capabilities. For instance, L162P, predicted to cause the most significant destabilization, is located in helix D, a region known to be critical for receptor interaction (as seen in previous contact analysis). The introduction of a proline residue in a helical region can often disrupt the helix structure, leading to instability. Conversely, the prediction that some mutations increase protein stability (e.g., S79C, D116E, T123M, C182R, C189Y) is also interesting. While destabilizing mutations are often linked to loss-of-function phenotypes, increased stability could potentially affect the dynamics of the protein or its ability to undergo conformational changes necessary for receptor activation [1,55].

It is important to note that changes in stability are just one aspect of how mutations can affect protein function. Other factors, such as changes in surface charge, hydrophobicity, or specific interactions with the receptor, can also play significant roles. For example, the R16C mutation, previously noted to reduce secretion, is predicted to be destabilizing by the SDM tool. Similarly, the R103C mutation in Kowarski syndrome, reported to have no effect on GHR signaling but stronger interaction with GHBP, shows a slightly destabilizing trend in this SDM analysis. Integrating the SDM analysis results with our previous findings on sequence conservation (ConSurf analysis) and known disease-causing mutations can provide a more comprehensive understanding of the molecular basis of growth hormone deficiencies. For example, mutations occurring in highly conserved residues that are also predicted to significantly destabilize the protein might be strong candidates for causing severe phenotypes [56].

By integrating the structural contact maps with thermodynamic stability predictions, a distinct genotype-phenotype correlation emerges, allowing us to categorize *GH1* mutations into three mechanistic classes.

**Class I: Folding and Secretory Competence.** Mutations such as L162P and L45P map to the hydrophobic core or helical scaffolding and are associated with high destabilization scores. These variants likely induce the unfolded protein response, preventing secretion. This aligns with clinical observations of extremely low serum GH levels in patients carrying these alleles.

**Class II: Primary Affinity Defects.** Variants such as K172N and F25Y cluster at the high-affinity Site 1 interface. These mutations typically show marginal impact on overall protein stability (ΔΔG near zero) but disrupt critical electrostatic or hydrophobic contacts with GHR Chain B. Phenotypically, these would present as GHD despite the presence of immunoreactive protein, as the hormone is incompetent for receptor capture.

**Class III: Dimerization and Signaling Blockade.** The most insidious class involves mutations at the Site 2 interface, such as G120C and D116E. These variants preserve the protein’s fold and its ability to bind the first receptor subunit, allowing for normal secretion and half-life. However, by sterically hindering the recruitment of the second GHR chain, they create a signaling-dead complex. This provides a comprehensive molecular explanation for Kowarski syndrome, where patients exhibit the paradox of short stature and low IGF-1 despite normal or elevated levels of circulating growth hormone.

Understanding the specific impact of each mutation on the two-site binding mechanism will contribute significantly to our knowledge of the molecular pathogenesis of growth disorders and may inform the development of targeted therapies. Further experimental studies by in vitro mutagenesis and functional assays, would be needed to validate the predictions and fully elucidate the impact of these mutations on growth hormone function and their association with growth disorders.

## 5. Conclusions

We have performed an analysis of the mutations in human growth hormone by combining structural, evolutionary, and clinical data. The study highlights key amino acids that are essential for GH to bind to GHR, a crucial interaction for growth and metabolic processes. These amino acids are conserved across various vertebrate species, emphasizing their significance in hGH function. We also found that disease-causing mutations frequently occur at these critical amino acid sites, disrupting the growth hormone’s ability to bind to its receptor and leading to growth disorders. The findings of this study offer a valuable framework for understanding the workings of human GH and the molecular underpinnings of growth hormone deficiency.

## Figures and Tables

**Table 1:** Sequence comparison of human growth hormone with growth hormone sequences from other species. The table shows the NCBI and UniProt sequence identifiers, UniProt sequence name, sequence length, percentage of sequence identity, percentage of sequence similarity, and signal peptide length for each species compared to human GH.

Organism	NCBI Seq ID	Uniprot Seq. ID	Uniprot Seq. Name	Seq Length	Seq Identity %	Seq Similarity %	Signal Pep
Human	NP_000506	P01241	SOMA_HUMAN	217	100	100	26
Rhesus macaque	NP_001036203	P33093	SOMA_MACMU	217	96	97	26
Rat	NP_001030020	P01244	SOMA_RAT	216	65	76	26
Mouse	NP_032143	P06880	SOMA_MOUSE	216	67	77	26
Horse	NP_001075417	P01245	SOMA_HORSE	216	67	79	26
Pig	NP_999034	P01248	SOMA_PIG	216	68	78	26
Bovine	NP_851339	P01246	SOMA_BOVIN	217	67	77	26
Sheep	NP_001009315	P67930	SOMA_SHEEP	217	67	76	26
Guinea pig	NP_001166330	Q9JKM4	SOMA_CAVPO	216	65	77	26
Common turkey	XP_010722827	P22077	SOMA_MELGA	216	55	73	25
Chicken	NP_989690	P08998	SOMA_CHICK	214	57	74	25
Common ostrich	BAA82959	Q9PWG3	SOMA_STRCA	215	54	72	25
Japanese eel	AAA48535	P08899	SOMA_ANGJA	207	44	61	19
Goldfish	AAC19389	O93359	SOMA1_CARAU	210	38	58	22
Atlantic salmon	AAU11454	Q5SDS1	Q5SDS1_SALSA	208	36	52	22

**Table 2:** A List of Disease-Causing Mutations in Human Growth Hormone. The table includes the amino acid change (Original Amino Acid → New Amino Acid), the sequence position in the full-length protein, the associated growth hormone deficiency type or syndrome, the reported effect of the mutation on GH function, the dbSNP identifier (if available), and the relevant publications. Sequence positions might differ depending on whether the signal peptide is included (residues 1–26). The table indicates both the position in the full-length protein and, where available from the source document, the position in the mature secreted growth hormone.

Growth Hormone Deficiency, Isolated, 1B (IGHD1B)					
Natural Variant	Sequence Position	PDB No.	Effect of Mutation	dbSNP	Publication
L → P	16	-	suppresses secretion		[49] Millar
D → N	37	11	-		[49] Millar
R → C	42	16	reduced secretion	rs71640273	[49] Millar
T → I	53	27	reduced ability to activate the JAK/STAT pathway		[49] Millar
K → R	67	41	reduced ability to activate the JAK/STAT pathway		[49] Millar
N → D	73	47	reduced ability to activate the JAK/STAT pathway	rs71640276	[49] Millar
S → F	97	71	reduced ability to activate the JAK/STAT pathway		[49] Millar
E → K	100	74	-		[49] Millar
Q → L	117	91	reduced secretion	Q → R	[49] Millar
S → C	134	108			[49] Millar
S → R	134	108	reduced ability to activate the JAK/STAT pathway		[49] Millar
T → A	201	175	reduced ability to activate the JAK/STAT pathway		[49] Millar
**Growth hormone deficiency, isolated, 2 (IGHD2)**					
R → H	209	183		rs137853223	[50] Miyata [51] Deladoey
**Kowarski syndrome (KWKS)**					
R → C	103	77	No effect on GHR signaling pathway; does not affect interaction with GHR; results in a stronger interaction with GHBP; does not affect the subcellular location.	rs137853220	[52] Takahashi [53] Petkovic
D → G	138	112	Loss of activity	rs137853221	[54] Takahashi

**Table 3:** A summary of amino acid contacts between human growth hormone (GH) and its receptor (GHR) (chains B and C). The table lists the GH residue (Mutated Protein Residue), its position (AA Pos), the corresponding residue in GHR chain B (Ref Prot Residue), the PDB residue number, the ConSurf conservation score of the GH residue, whether it was identified as a contact residue in LigPlot+ analysis for chain B (Contact R 1) and chain C (Contact R 2), and if it is a known disease variant according to UniProt (UniProt Disease Variant).

Clinical Significance	Protein Residue	AA Pos	Ref Prot Res	PDB Res	ConSurf Conservation	Contact R 1	Contact R 2	UniProt Disease Variant
	Gln [Q]	28	Pro [P]	2	e		Yes (2)	
	Thr [T]	30	Ile [I]	4	b		Yes (4)	
	Lys [K]	34	Arg [R]	8	e		Yes (8)	
	His [H]	38	Asn [N]	12	e		Yes (12)	
	Phe [F]	41	Leu [L]	15	b		Yes (15)	
	His [H]	42	Arg [R]	16	e, f		Yes (16)	Yes (R>C)
	Thr [T]	43	Ala [A]	17	b, s			
	Arg [R]	44	His [H]	18	e	Yes (18)		
	Tyr [Y]	47	His [H]	21	e, f	Yes (21)		
	Thr [T]	50	Ala [A]	24	b, s			
B	Tyr [Y]	51	Phe [F]	25	e	Yes (25)		
	Pro [P]	71	Leu [L]	45	e	Yes (45)		
	Lys [K]	73	Asn [N]	47	e			Yes (N>D)
	Thr [T]	74	Pro [P]	48	e	Yes (48)		
P	Ser [S]	79	Cys [C]	53	b, s			
	Cys [C]	88	Ser [S]	62	e	Yes (62)		
	Lys [K]	89	Asn [N]	63	e	Yes (63)		
	His [H]	103	Arg [R]	77	e			Yes (R>C)
P	Cys [C]	103		77				
	Cys [C]	105	Ser [S]	79	b, s			
	Glu [E]	110	Gln [Q]	84	e, f			
	Arg [R]	117	Gln [Q]	91	e			Yes (Q>L)
P	Gly [G]	138	Asp [D]	112	e			Yes (D>G)
	Glu [E]	142	Asp [D]	116	e		Yes (116)	
	Asp [D]	145	Glu [E]	119	e		Yes (119)	
	Ser [S]	146	Gly [G]	120	b		Yes (120)	
	Met [M]	149	Thr [T]	123	b		Yes (123)	
	Pro [P]	188	Leu [L]	162	b, s			
	His [H]	190	Tyr [Y]	164	b	Yes (164)		
	Glu [E]	195	Asp [D]	169	e, f			
	Asn [N]	198	Lys [K]	172	e, f	Yes (172)		
	Lys [K]	200	Glu [E]	174	e	Yes (174)		
	Met [M]	205	Ile [I]	179	b	Yes (179)		
	Arg [R]	208	Cys [C]	182	b, s	Yes (182)		
P	His [H]	209	Arg [R]	183	e			Yes (R>H)
	Tyr [Y]	215	Cys [C]	189	e, f	Yes (189)		
	Ser [S]	216	Gly [G]	190	e	Yes (190)		

**Table 4:** ConSurf amino acid conservation scores for selected mutations in the human growth hormone protein. Conservation scores range from 1 (variable) to 9 (highly conserved). The residue variety across species indicates the different amino acids observed at that position in orthologous sequences. Higher conservation scores are shown in red.

**SNV**	**Conservation Score**	**Residue Variety Across Species**
P2Q	8	P,Y,V
I4T	5	A,F,T,P,E,V,M,I,L
R8K	4	S,W,N,K,E,H,Q,D,R,G
N12H	7	S,T,N,K,E,H,M,C,I,R,L
L15F	1	S,F,T,N,K,E,V,H,Q,M,R,I,G,L
R16H, R16L, R16C	7	H,Q,R,Y,L,V
A17T	8	S,A,T,I,L,V
H18R	7	S,W,T,N,E,H,Q,D
H21Y	7	F,S,H,K,R,Y,V
A24T	8	S,A,T,N,Y,V
F25Y, F25I	6	S,A,F,T,K,E,Y,Q,M,D,R,I,G,L
L45P	6	L
N47K, N47D	3	S,A,T,N,P,K,V,H,M,D,I,G
C53S, C53F	9	C
S62C	5	A,S,T,N,K,E,V,H,Q,M,I,G
N63K	7	S,D,N,P,G,E
R77H, R77C	5	S,N,K,H,Q,D,R,G,L
S79C	7	A,S,M,T,I,G,V
Q84F	6	S,W,P,Y,E,V,H,Q,M,D,R,I,L
Q91R	3	S,F,A,N,K,E,Y,V,H,Q,D,R,I,G,L
D112G, G112H	1	A,S,T,N,K,P,E,H,Q,D,R,G,L
D116E, D116N	4	A,S,N,K,E,Y,V,Q,D,R,I,G
E119D	4	S,A,T,N,K,E,V,Q,M,D,R,L
G120S, G120C	9	A,F,T,G,Y
T123M	2	S,A,T,N,K,E,V,M,R,I,L
L162P	7	F,T,M,N,K,I,L,V
Y164H	6	A,S,T,N,Y,H,M,C,R
D169E	9	D,E
K172N	9	H,M,N,R,K
E174K	9	S,Q,D,Y,E
I179V, I179M, I179S	7	F,T,M,I,L,V
C182R	9	C
R183H, R183C	9	Q,K,R
C189Y	9	C
G190S	4	S,A,T,G

**Table 5:** Impact of amino acid changes caused by human genetic variations on GH residues involved in contact with GHR. Amino acids changes reported at positions identified as involved in binding to GHR were collected from NCBI genomic database. After making a contact map of GH-GHR interactions, all amino acid variations reported in GH1 gene were searched and variations occurring at amino acids of GH making contact with GHR were further analyzed by virtual site direct mutagenesis tool (SDM). Predicted changes in protein stability (ΔΔG) selected mutations in the human growth hormone protein, as analyzed by the SDM tool. The table includes the mutated residue (Mutation), secondary structure element (SSE) and relative solvent accessibility (RSA) for both the wild-type (WT) and mutant (MT) proteins, residue depth (DEPTH), outer shell potential (OSP), structural class (SS), structural neighborhood (SN), and solvent organization (SO). The predicted ΔΔG (kcal/mol) indicates the change in protein stability upon mutation, with a positive value suggesting decreased stability and a negative value suggesting increased stability. The Stability column indicates whether the mutation is predicted to destabilize (-) or stabilize (+) the protein.

Mutation	WT_SSE	WT_RSA (%)	WT_DEPTH ( Å )	WT_OSP	WT_SS	WT_SN	WT_SO	MT_SSE	MT_RSA (%)	MT_DEPTH ( Å )	MT_OSP	MT_SS	MT_SN	MT_SO	Predicted ΔΔG	Stability
P2Q	p	89	3.2	0.11	-	-	-	p	99	3.3	0.08	-	-	-	−0.8	-
I4T	b	55.1	3.5	0.33	-	-	-	b	70.7	3.4	0.26	-	-	-	−1.09	-
I4V	b	55.1	3.5	0.33	-	-	-	b	55.1	3.3	0.33	-	-	-	−0.07	-
R8K	H	59.6	3.4	0.31	+	-	-	H	66.7	3.5	0.27	-	-	-	−0.43	-
N12H	H	57.8	3.5	0.33	+	-	+	H	58.6	3.5	0.26	+	-	-	0.68	+
L15F	H	74.6	3.2	0.23	-	-	-	H	79.7	3.3	0.19	-	-	-	−0.63	-
R16H	H	44.2	3.8	0.34	+	-	-	H	31.9	3.9	0.4	+	-	+	0.19	+
R16L	H	44.2	3.8	0.34	+	-	-	H	20.9	4.1	0.44	-	-	-	0.39	+
R16C	H	44.2	3.8	0.34	+	-	-	H	22.1	4.2	0.44	+	-	+	−0.76	-
A17T	H	1.4	6.7	0.54	-	-	-	H	0.3	6.9	0.59	-	+	-	−1.88	-
H18R	H	69.2	3.4	0.26	+	-	-	H	70.1	3.4	0.2	-	-	-	0.06	+
H21Y	H	18.8	4.3	0.47	-	-	-	H	25.8	4.5	0.41	-	-	-	0.65	+
A24T	H	0	8.3	0.57	-	-	-	H	0	8.4	0.65	-	-	+	−3.21	-
F25Y	H	57.4	3.6	0.27	-	-	-	H	57.6	3.6	0.27	-	-	-	0.47	+
F25I	H	57.4	3.6	0.27	-	-	-	H	47.8	3.6	0.34	-	-	-	0.31	+
L45P	H	40.9	3.6	0.33	-	-	-	H	34	3.7	0.33	-	-	-	−2.23	-
P48T	H	78	3.1	0.2	-	-	-	H	95	3.2	0.16	-	-	-	0.34	+
C53S	b	3.7	5.9	0.43	+	-	+	b	4.5	5.8	0.42	+	-	-	−1.11	-
N47K	b	62.6	3.3	0.39	+	+	+	b	82.8	3.3	0.2	-	-	-	−0.32	-
N47D	b	62.6	3.3	0.39	+	+	+	b	67.5	3.3	0.33	-	-	+	−0.44	-
C53F	b	3.7	5.9	0.43	+	-	+	b	3.6	5.2	0.51	-	-	-	−0.62	-
S62C	a	84.6	3.1	0.14	+	-	-	a	90.8	3.2	0.11	-	-	-	0.62	+
N63K	b	71.2	3.5	0.3	+	+	-	b	83.5	3.3	0.16	-	-	-	−0.18	-
R77H	H	19.4	4.9	0.45	-	-	+	H	17	4.7	0.54	+	-	+	−0.07	-
R77C	H	19.4	4.9	0.45	-	-	+	H	12.1	5	0.57	-	-	+	−0.71	-
S79C	H	0	10.9	0.57	-	-	+	H	0	10.8	0.61	-	+	+	1.52	+
Q84E	H	21.4	4	0.43	+	-	-	H	12.1	4.4	0.45	+	-	-	0.4	+
Q91R	H	64.4	3.4	0.24	-	-	-	H	78.7	3.4	0.16	-	-	-	−0.15	-
Q91K	H	64.4	3.4	0.24	-	-	-	H	57.2	3.4	0.21	-	-	-	−0.44	-
Q91L	H	64.4	3.4	0.24	-	-	-	H	62.9	3.4	0.23	-	-	-	0.29	+
D112G	H	78.3	3.4	0.27	-	-	+	H	82.6	3.7	0.34	-	-	-	−0.16	-
D112H	H	78.3	3.4	0.27	-	-	+	H	76.7	3.4	0.25	-	-	+	0.88	+
D116E	H	54.7	3.6	0.31	+	-	-	H	57.1	3.8	0.26	-	-	-	1.25	+
D116N	H	54.7	3.6	0.31	+	-	-	H	61.9	3.7	0.29	+	-	-	−0.35	-
E119D	H	85.5	3.3	0.2	-	-	-	H	80.4	3.3	0.25	-	-	-	−1.48	-
G120S	H	64.2	4.6	0.46	-	-	-	H	30.8	4.2	0.42	-	-	+	0.18	+
G120C	H	64.2	4.6	0.46	-	-	-	H	30.1	4.2	0.4	-	-	+	0.7	+
T123M	H	47.5	3.8	0.29	-	-	+	H	52.2	3.6	0.24	-	-	-	1.19	+
L162P	H	3.5	6	0.48	-	-	-	H	16.4	5.4	0.39	-	-	-	−4.31	-
Y164H	H	13.3	5.2	0.48	-	-	-	H	10.8	4.9	0.48	-	-	-	−1.27	-
D169E	H	1.4	7.7	0.53	+	-	+	H	3	9.3	0.6	+	-	+	−0.01	-
K172N	H	27.5	3.9	0.4	-	-	-	H	35	4.3	0.42	-	-	+	−0.69	-
E174K	H	24.3	3.7	0.4	+	-	-	H	32.8	4.2	0.33	-	-	-	−1.01	-
I179M	H	23	4.2	0.4	-	-	-	H	32.8	4.1	0.32	+	-	-	−0.02	-
I179S	H	23	4.2	0.4	-	-	-	H	19.5	4.5	0.39	-	-	+	−0.8	-
I179V	H	23	4.2	0.4	-	-	-	H	20.4	4.3	0.43	-	-	-	−0.35	-
C182R	H	28.7	3.5	0.4	+	-	+	H	68.5	3.6	0.21	-	-	-	1.04	+
R183H	H	31.8	3.7	0.3	+	-	+	H	77.5	3.4	0.16	-	-	-	−1.1	-
R183C	H	31.8	3.7	0.3	+	-	+	H	72.9	3.2	0.2	-	-	-	−0.65	-
C189Y	a	22.9	3.9	0.29	+	-	-	a	75.2	3.4	0.11	-	-	-	0.78	+
G190S	b	199.3	3.5	0.07	-	-	-	p	107.2	3.1	0.08	-	-	-	0	+

## Data Availability

The original contributions presented in this study are included in the article. Further inquiries can be directed to the corresponding author.

## Abbreviations

The following abbreviations are used in this manuscript:

GH	Growth hormone
GHR	Growth hormone receptor
GHD	Growth hormone deficiency
GHBP	Growth hormone binding protein
